# Supplementary material for: Neutralizing Activity against BQ.1.1, BN.1, and XBB.1 in Bivalent COVID-19 Vaccine Recipients: Comparison by the Types of Prior Infection and Vaccine Formulations
Source: Vaccines (Basel). 2023 Aug 4;11(8):1320. doi: 10.3390/vaccines11081320 (PMC10458764; doi:10.3390/vaccines11081320)
Supplement: Supplementary file 1 [file vaccines-11-01320-s001.zip › Final_Table S1_vaccines.pdf]

Table S1. Comparison of IgG anti-RBD antibodies after bivalent booster: comparison by the presence of prior SARS-CoV-2 infection

	Group 1	Group 2	Group 3	*P*-value		
	**SARS-CoV-2 infection-naïve (n = 5)**	**Prior BA.1/BA.2- infected (n = 10)**	**Prior BA.5- infected (n = 6)**	**Group 1 vs. Group 2**	**Group 2 vs. Group 3**	**Group 3 Vs. Group 1**
Pre-bivalent vaccine GMT, U/mL	1650 (607–4484)	9675 (7006–13,360)	20,233 (6121–66,881)	0.003	0.042	0.009
Post-bivalent vaccine GMT, U/mL	29,303 (19,129–44,887)	39,043 (31,333–48,650)	44,762 (28,593–70,075)	0.165	0.511	0.247

Abbreviations: IgG, immunoglobulin G; RBD, receptor binding domain, GMT, geometric mean titer.
