# Supplementary figures and images for: Neutralizing Activity against BQ.1.1, BN.1, and XBB.1 in Bivalent COVID-19 Vaccine Recipients: Comparison by the Types of Prior Infection and Vaccine Formulations
Source: Vaccines (Basel). 2023 Aug 4;11(8):1320. doi: 10.3390/vaccines11081320 (PMC10458764; doi:10.3390/vaccines11081320)

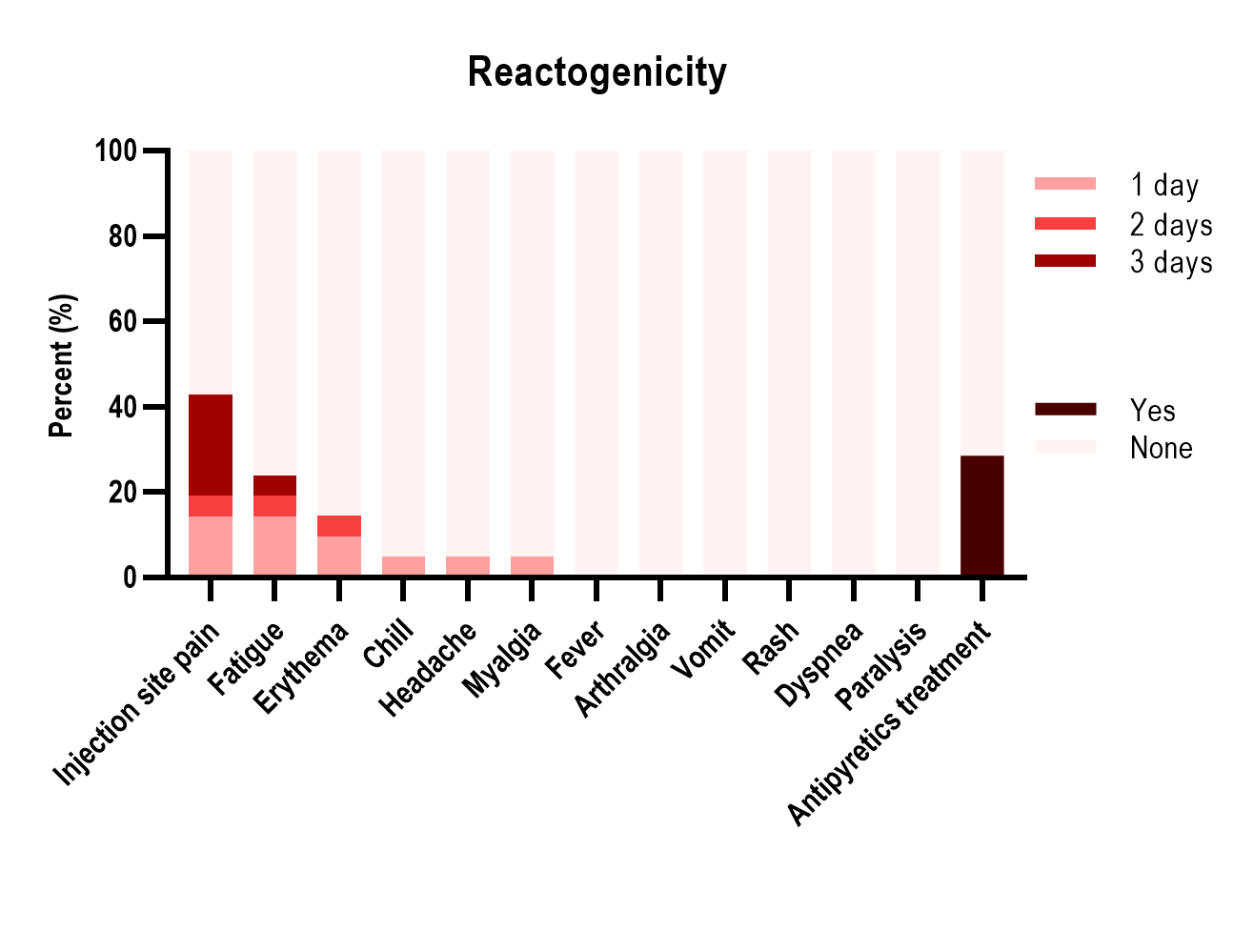

Supplement: Supplementary file 1 [file vaccines-11-01320-s001.zip › Figure S1.tif]
